# A challenging case of pregnancy with placenta accreta and very rare irregular antibodies versus Cromer blood group system: a case report

**DOI:** 10.1186/s13256-015-0607-7

**Published:** 2015-05-15

**Authors:** Stefano Busani, Annamaria Ghirardini, Elisabetta Petrella, Isabella Neri, Federico Casari, Donatella Venturelli, Mario De Santis, Giuliano Montagnani, Fabio Facchinetti, Massimo Girardis

**Affiliations:** 1 Cattedra e Servizio di Anestesia e Rianimazione 1, Azienda Ospedaliera Universitaria Policlinico di Modena, Modena, Italy; 2 Reparto di Ostetricia e Ginecologia, Azienda Ospedaliera Universitaria Policlinico di Modena, Modena, Italy; 3 Servizio di Radiologia 1, Azienda Ospedaliera Universitaria Policlinico di Modena, Modena, Italy; 4 Servizio Immunotrasfusionale, Azienda Ospedaliera Universitaria Policlinico di Modena, Modena, Italy

**Keywords:** Cromer blood group system antibodies, Endovascular intervention, Intensive care unit, Peripartum hemorrhage, Placenta accreta

## Abstract

**Introduction:**

This report describes the challenges of treating a pregnant woman who had a rare case of critical placenta accreta with concurrent Cromer system anti-Tc(a) and anti-Kidd A alloantibodies. No previous case of such alloimmunization in a patient with placenta accreta has been reported.

**Case presentation:**

A 28-year-old African woman with anti-Cromer Tc(a) antibodies, anti-Kidd A antibodies and placenta accreta was admitted to the obstetric emergency department at our university hospital with persistent vaginal bleeding. Her rare Cromer blood group system antibodies had been diagnosed 1 month earlier; no compatible blood had been found despite a worldwide search. We performed a cesarean section after placement of Fogarty balloons in her uterine arteries with preoperative endovascular interventional radiology. Other therapeutic interventions included preoperative iron administration to raise hemoglobin and the scheduled predeposit of autologous blood. Intraoperative therapeutic management was aimed at preventing coagulopathy and massive bleeding. With the use of alternative medical techniques determined during perioperative planning, her intraoperative blood loss was only 1000mL, despite the placenta accreta. She was discharged from the hospital 4 days after cesarean section.

**Conclusions:**

To the best of our knowledge, this is the first report of an alloimmunized patient with two different alloantibodies and concurrent high risk of bleeding because of placenta accreta. The close collaboration among obstetricians, anesthesiologists, interventional radiologists, blood bank pathologists and intensive care doctors prevented serious consequences in this patient. The exceptional feature of this case is the patient’s double risk: the placenta accreta and the inability to transfuse compatible blood. These two extreme situations challenged the multidisciplinary medical team.

## Introduction

The Italian National Institute of Health recently conducted a study to analyze causes of maternal death and to compute maternal mortality ratios in six regions of Italy, including our region. The total maternal mortality ratio was 11.8/100,000 live births, with hemorrhage and hypertensive disorders the greatest risk factors for obstetric death [1]. The management of pregnant women with a high risk of bleeding remains a great clinical challenge, requiring the cooperation of a multidisciplinary medical team. Clinicians should engage in a detailed discussion to anticipate possible complications during childbirth, and to define the optimal time of delivery, location of procedures, sequence of events, transfusion requirements, type of anesthesia and postoperative care [2-4].

Patients with abnormal placental insertion, including placenta accreta, increta and percreta, have a high risk of hemorrhage. These conditions are characterized by abnormally tight adhesion between placenta and uterus, which can result in massive hemorrhage during delivery [5]. In patients with blood group abnormalities, such as the presence of rare antibodies against the Cromer blood group system, the risk of death during delivery becomes exponentially higher [6-8].

We report the first case of a pregnant woman with critical placenta accreta and concurrent Cromer anti-Tc(a) antibodies and anti-Kidd A (JKa) antibodies. Because of her high risk of hemorrhage and the extreme difficulty of finding compatible blood, the patient had a cesarean delivery with the intraoperative support of a multidisciplinary team.

## Case presentation

A 28-year-old African woman (72kg, 166cm) with anti-Cromer Tc(a) and anti-Jka antibodies was admitted to the obstetric emergency department at our University Hospital at 31+4 weeks’ gestation for heavy vaginal bleeding. She had a history of two prior at-term cesarean deliveries (in 2006 and 2010) and two voluntary abortions. She had a positive indirect Coombs test near the beginning of pregnancy. Low-titer anti-Tc(a) antibodies had been identified and closely monitored.

A month before admission to our department (at 27+3 weeks’ gestation), she had been hospitalized for cervical shortening (23mm). Ultrasonography at that time had revealed full placenta previa and a high risk for placenta accreta. In accordance with the guidelines of the Royal College of Obstetricians and Gynaecologists [9], the ultrasound report was as follows: “… in grey-scale, a thinning of the hyperechoic serosa–bladder interface and abnormal placental lacunae were found. Moreover, the placental tissue on the left side of the uterus appeared to reach the serosa.” During the initial hospitalization, two injections of betamethasone (12mg) were administered to prevent fetal respiratory distress, and a consulting hematologist requested further immunohematological testing.

Because of the rarity of antibodies of the Cromer blood group system and the high risk of heavy bleeding associated with placenta accreta, it was necessary to establish specific policies for the planned delivery by cesarean section. In preparation, parenteral iron supplementation was administered twice weekly beginning in the 28th week to raise hemoglobin (Hb) values above 12g/dL from an initial Hb of 10.5g/dL. Meanwhile, the Transfusion Medicine Department consultant planned two autologous blood donations because no compatible blood was found in a search of the United States Blood Bank network or the network of European blood banks (World Health Organization International Rare Donor Panel). Unfortunately, the autologous blood could not be collected because of sudden worsening of the patient’s clinical condition.

At her readmission to our Obstetrical Emergency Department, serial ultrasound examinations and fetal monitoring with cardiotocography were performed. Because vaginal bleeding stopped within a few hours in response to tocolytic therapy, the attending physicians decided to maintain a conservative strategy. Elective cesarean delivery was scheduled with multidisciplinary team cooperation because of the extreme complexity of the case. The cesarean section was performed 3 days later, after a long and detailed discussion among anesthesiologists, obstetricians, radiologists, hematologists and intensivists. Her preoperative Hb was 10.2g/dL.

**Figure 1:**
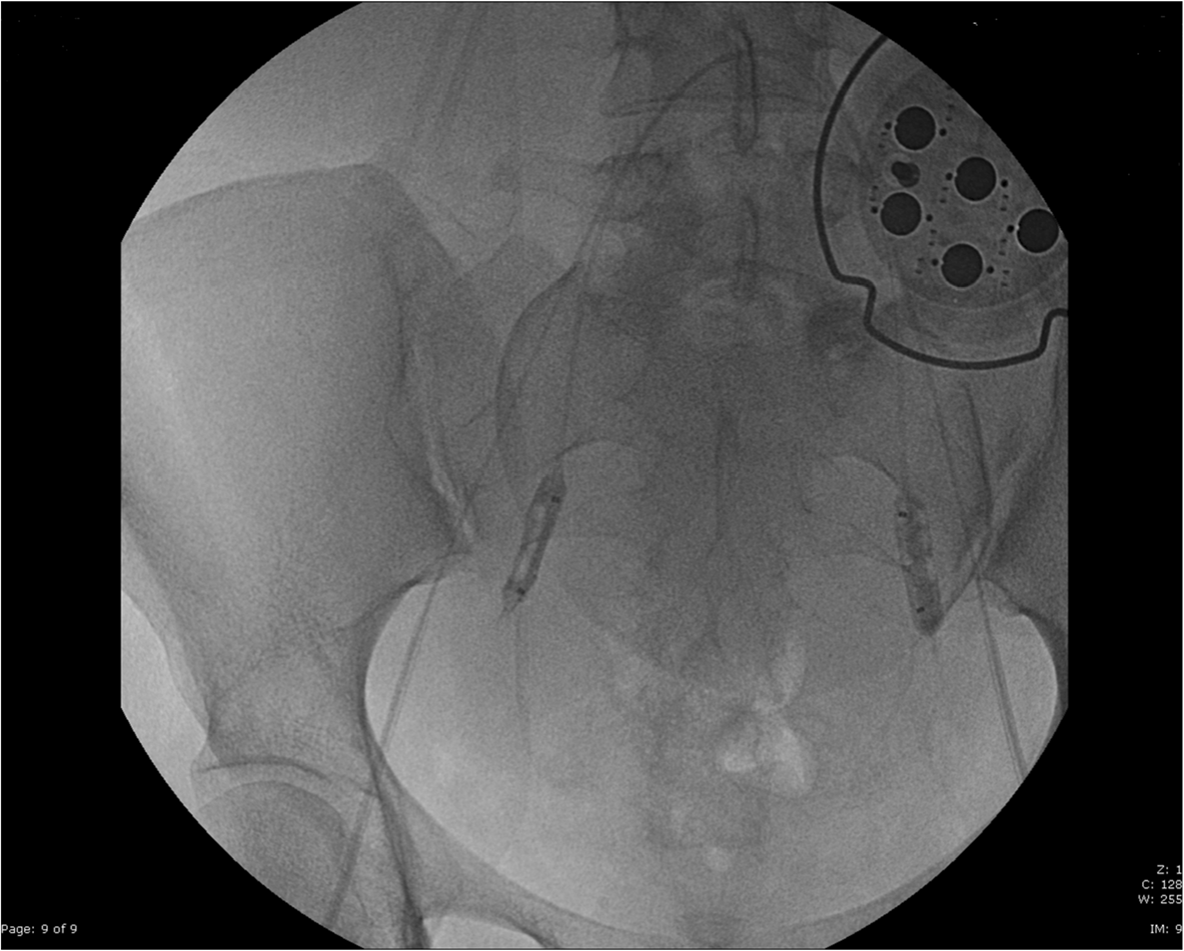
The two Fogarty balloons, over the distal tips of the 5 Fr sheaths, inside the uterine arteries. Both balloons were inflated for the confirmation of the effectiveness of the stop flow through fluoroscopic image.

Before cesarean section, the patient provided written informed consent and was brought to the angiography suite (Philips Medical Systems, Best, Netherlands). We placed two 14-gauge and one 16-gauge peripheral venous catheters and performed radial artery cannulation to monitor intra-arterial pressure throughout the procedures. After local anesthesia of her groin bilaterally, her femoral arteries were punctured with Seldinger technique and 4-Fr sheaths (Terumo, Leuven, Belgium) were placed to allow bilateral cross catheterization of her hypogastric arteries with a C1 “Cobra” catheter (Terumo Leuven, Belgium). Using a 0.035 inch 150cm Amplatz guidewire (Cordis, Fremont, CA, USA), the 4-Fr sheaths were exchanged with two 45cm 5-Fr Brite Tip® sheaths (Cordis, Fremont, CA, USA), with the distal tips in the anterior branches of both hypogastric arteries. This allowed placement of two Fogarty 80cm 4-Fr balloons over the distal tips of the 5-Fr sheaths, within her uterine arteries. Proper positioning of the Fogarty balloons and the effectiveness of vascular occlusion were confirmed with fluoroscopy (Figure 1). Maternal and fetal monitoring were conducted throughout the procedure, without complications.

She was transferred from the angiography suite to the obstetric operating room. A single fluoroscopic image was made with a moveable C-arm to confirm that the Fogarty balloons remained in the proper position. While she underwent the angiographic procedure, the operating room was set up with an intraoperative blood salvage system (Cell Saver®; Haemonetics Corp., Braintree, MA, USA), a rapid infusion system (Haemonetics Corp., Braintree, MA, USA), fluid warmers (Level 1 H-1200; Smiths Medical, Kent, UK) and forced air warming systems (Bair-Hugger™, 3M, St. Paul, MN, USA). Before anesthetic induction, she received premedication with hydrocortisone (200mg) as recommended by the hematologist in case of emergent need to transfuse incompatible blood. She also received antibiotic prophylaxis and intravenous tranexamic acid (750mg) to prevent peripartum hemorrhage [10]. Anesthesia was induced with 200mg propofol, 200mcg fentanyl and 40mg rocuronium and was maintained with sevoflurane gas. A low transverse skin incision through her previous cesarean scars and a lower uterine segment incision were performed, avoiding the upper margin of the anterior placenta. Immediately after delivery of the fetus and umbilical cord clamping, the uterine artery balloons were inflated. As per the obstetrician’s request, the uterine arteries were embolized with Gelfoam® sponge (Jinling Pharmaceutical Company, Nanjing, China) to stop bleeding. Because the placenta could not be completely removed as a result of its strong adhesion to the posterior wall of the uterus, and because of the risk of postoperative hemorrhage, the surgeons decided to perform a total hysterectomy, as recommended by Royal College of Obstetricians and Gynaecologists guidelines [9]. When adequate hemostatic control was achieved, the balloons were deflated. After the uterine lumen and abdominal cavity were closed and no vaginal bleeding was confirmed, the Fogarty balloons were removed. The 5-Fr sheaths were maintained for 24 hours in case further embolization was necessary. The total intraoperative blood loss was 1000mL and the surgery lasted approximately 150 minutes. During the cesarean section, 150mL of blood was reinfused through the cell salvage technology and limited crystalloid fluid therapy was administered (1L of lactated Ringer’s solution). Her temperature was maintained at approximately 37°C throughout the procedure and serial blood gas analyses were performed to monitor for metabolic acidosis and electrolyte abnormalities. Further details of our hemorrhage management protocol have been reported previously [11].

After surgery, she was admitted to our Intensive Care Unit (ICU) for close monitoring of bleeding and coagulation profiles. Immediately after ICU admission, ventilation was optimized, adequate analgesia was provided and she was awakened and extubated. Because no further bleeding was recorded from the abdominal drain, the angiographic 5-Fr sheaths were removed the day after surgery and she was transferred to the obstetric ward with a Hb of 8.9g/dL.

Four days after cesarean section, she was discharged from our hospital with a prescription for oral iron therapy for at least 30 days and plans for blood count recheck 15 days after discharge.

## Discussion

The presence of antibodies against the Cromer blood group system is a rare hematologic condition, reported only a few times, and thus this condition is probably underdiagnosed [6-8]. Diagnosis is important because appropriate hematologic, anesthetic, radiologic and intensive care support is necessary in case of surgery for individuals with this condition. Individuals (only nine identified by the World Health Organization International Rare Donor Panel to date) negative for all Cromer antigens (null phenotype) lack the decay-accelerating factor (DAF) on their red blood cells (RBC) but do not have an increased susceptibility to hemolysis during normal activities of daily living [8]. The antigens (12 high- and three low-prevalence) of the Cromer blood group system are carried on DAF, which is a member of the family of complement activation regulators. One function of DAF is to protect cells from autologous complement attack [12]. DAF is expressed on RBC, white blood cells, platelets and epithelial tissues, and on the apical surface of placental trophoblasts, which are thought to filter out antibodies. This suggests that DAF may play a strategic role in protecting the antigenically foreign fetus from maternal complement attack, thus performing an important role in the maintenance of human pregnancy [8]. Antibodies with specificity to the Cromer system antigens, anti-Tc(a), can cause post-transfusion severe hemolytic reactions [6,7]. Pregnancy is definitely a risk for Cromer-sensitized patients and can be life-threatening if associated with obstetric anomalies that predispose to massive hemorrhage.

Several medical professionals were included in the long discussion that was held before the cesarean section, focusing on a series of questions. Every possible strategy to prevent massive bleeding was debated at length. It was agreed that endovascular intervention with temporary balloons was necessary and that hysterectomy would be performed if critical bleeding occurred. Despite the lack of strong evidence for its use [13], some centers have established protocols for interventional radiology to reduce blood loss in cases of placenta accreta. We decided to pursue this strategy, based on the experience of our interventional radiologists. Although hysterectomy is recommended only in case of massive bleeding, the procedure was performed in this case to prevent possible further postoperative bleeding.

Another critical point that we needed to address was the patient’s intraoperative management, including monitoring, fluids, procoagulant drugs and type of anesthesia. We decided to perform general anesthesia with the intraoperative use of cell salvage technology, despite the strongly debated risk of amniotic fluid embolism [14]. Because we had no other option for transfusing this patient, we decided to assume this risk; fortunately amniotic fluid embolism did not occur. During the cesarean section, the coagulation system was the focus of attention to avoid triggering coagulopathy; the patient’s temperature, acid-base balance and electrolytes were closely monitored and readily corrected. The most salient point in the long discussion among anesthesiologists and blood bank pathologists was the Hb cut-off for transfusion with incompatible blood. In the end, we decided to risk transfusing the patient if her Hb fell below 6g/dL. We were well aware of the risk of poor tissue perfusion if the cut-off was too low, and of severe hemolytic syndrome if the cut-off was too high.

## Conclusions

The exceptional feature of this case was the double risk to which this pregnant patient was subjected rather than the treatment she received. The presence of placenta accreta with no availability of compatible blood for transfusion challenged the multidisciplinary team. The close collaboration among obstetricians, anesthesiologists, interventional radiologists, blood bank pathologists and intensive care doctors prevented serious consequences in this patient. We believe that this case provides interesting ideas that may aid clinicians in treating patients with exceptional risk of bleeding and the inability to transfuse compatible blood as a result of alloimmunization. We believe that the choices we had to make will provide food for thought for those handling similar situations in the future.

## Consent

Written informed consent was obtained from the patient for publication of this case report and accompanying images. A copy of the written consent is available for review by the Editor-in-Chief of this journal.

## Abbreviations

Anti-Jka: Anti-Kidd A
DAF: Decay-accelerating factor
Hb: Hemoglobin
ICU: Intensive Care Unit
RBC: Red blood cells

## References

[1] Senatore S, Donati S, Andreozzi S. Studio della cause di mortalità e morbosità materna e messa a punto di modelli di sorveglianza della mortalità materna. Rapporti ISTISAN 12/6, 2012. Available on website: http://www.iss.it/binary/publ/cont/dodici6web.pdf

[2] Peralta F, Wong CA. Interventional radiology in the pregnant patient for obstetric and nonobstetric indications: organizational, anesthetic, and procedural issues. Curr Opin Anaesthesiol. 2013;26(4):450–5. 10.1097/ACO.0b013e3283625e89 23756912

[3] Snegovskikh D, Clebone A, Norwitz E. Anesthetic management of patients with placenta accreta and resuscitation strategies for associated massive hemorrhage. Curr Opin Anaesthesiol. 2011;24(3):274–81. 10.1097/ACO.0b013e328345d8b7 21494133

[4] Abdul-Kadir R, McLintock C, Ducloy AS, El-Refaey H, England A, Federici AB, et al. Evaluation and management of postpartum hemorrhage: consensus from an international expert panel. Transfusion. 2014;54(7):1756–68. 10.1111/trf.12550 24617726

[5] Riteau AS, Tassin M, Chambon G, Le Vaillant C, de Laveaucoupet J, Quéré MP, et al. Accuracy of ultrasonography and magnetic resonance imaging in the diagnosis of placenta accreta. PLoS One. 2014;9(4), e94866. 10.1371/journal.pone.0094866 PMC3986371 24733409

[6] Lublin DM. Review: Cromer and DAF: role in health and disease. Immunohematology. 2005;21(2):39–47. 15954803

[7] Storry JR, Reid ME, Yazer MH. The Cromer blood group system: a review. Immunohematology. 2010;26(3):109–18. 21214297

[8] Weber SL, Bryant BJ, Indrikovs AJ. Sequestration of anti-Cra in the placenta: serologic demonstration by placental elution. Transfusion. 2005;45(8):1327–30. 10.1111/j.1537-2995.2005.00205.x 16078921

[9] Placenta praevia, placenta praevia accreta and vasa praevia: diagnosis and management. http://www.rcog.org.uk/guidelines. Green-top Guideline No. 27. January 2011.

[10] Xu J, Gao W, Ju Y. Tranexamic acid for the prevention of postpartum hemorrhage after caesarean section: a double-blind randomization trial. Arch Gynecol Obstet. 2013;287(3):463–8. 10.1007/s00404-012-2593-y 23064441

[11] Busani S, Cavazzuti I, Marietta M, Pasetto A, Girardis M. Strategies to control massive abdominal bleeding. Transplant Proc. 2008;40(4):1212–5. 10.1016/j.transproceed.2008.03.119 18555150

[12] Hue-Roye K, Lomas-Francis C, Belaygorod L, Lublin DM, Barnes J, Chung A, et al. Three new high-prevalence antigens in the Cromer blood group system. Transfusion. 2007;47(9):1621–9. 10.1111/j.1537-2995.2007.01333.x 17725726

[13] Lee JS, Shepherd SM. Endovascular treatment of postpartum hemorrhage. Clin Obstet Gynecol. 2010;53(1):209–18. 10.1097/GRF.0b013e3181ce09f5 20142657

[14] Sullivan I, Faulds J, Ralph C. Contamination of salvaged maternal blood by amniotic fluid and fetal red cells during elective Caesarean section. Br J Anaesth. 2008;101(2):225–9. 10.1093/bja/aen135 PMC2447531 18515817

